# A Singular Case.—Extrusion of a Bicuspid

**Published:** 1879-12

**Authors:** 


### ARTICLE VI.

### *A Singular Case.—Extrusion of Bicuspid.*

BY PROF. HODGKIN.

Mr. C. brought in his little boy of five and half years, stating that he needed some extraction done. On examination it was seen that the six-year molars were erupted above and below. The crown of the first temporary molar left upper was gone, the roots remaining in position but loose. On the first casual examination I saw what I fancied was the buccal root of this tooth pushed out through the gum, as is seen occasionally when teeth are in process of eruption—the outcoming tooth pressing the root laterally out and through the process. But on a more carefu inspection it was seen that the appearance on the buccal aspect of the alveolar ridge was the *first bicuspid*; the crown fully formed but minus the root. This tooth was clinging slightly to the gum, by its lingual face; the upper or root aspect entirely exposed, and funnel shaped, as is usual in these partly developed cases. The adhesion to the gum was so slight, and its dislodgment from its socket so complete as to render only the finger nail necessary for its removal. The pulp was of course gone and the cavity empty. No examination of the jaw was allowed to discover if any growth had forced this tooth thus early from its place, though no evidence of trouble was present to sight. Why was this bicuspid thus thrust out of its socket laterally, and protruding wrong end foremost from the alveolus?

